# Factors associated with limitations in daily life and at work in a population with shoulder pain

**DOI:** 10.1186/s12891-022-05638-6

**Published:** 2022-08-15

**Authors:** Diane Godeau, Marc Fadel, Alexis Descatha

**Affiliations:** 1 Université Paris-Saclay, UVSQ, Univ. Paris-Sud, Inserm, Équipe d’Épidémiologie Respiratoire Intégrative, CESP, 94807 Villejuif, France; 2 AP-HP (“Assistance Publique-Hôpitaux de Paris”), Unité des pathologies professionnelles et environnementales, Hôpitaux universitaires Paris Seine-Saint-Denis, Hôpital Avicenne, F-93009 Bobigny cedex, France; 3 Université Sorbonne Paris Nord, F-93206 Saint-Denis, France; 4 Godeau, Hôpital Avicenne, Unité fonctionnelle des pathologies professionnelles et environnementales, 125 rue de Stalingrad, 93009 Bobigny cedex, France; 5 Univ Angers, CHU Angers, Univ Rennes, Inserm, EHESP, Irset (Institut de recherche en santé, environnement et travail) - UMR_S 1085, F-49000 Angers, France; 6 CHU Angers, Poisoning Control Center- Clinical Data Center, F-49000 Angers, France

**Keywords:** Disability, Limitations, Daily life, Work

## Abstract

**Background:**

Shoulder pain, which is a widespread condition, can lead to participation restrictions in daily and professional life. However, there are few studies focusing on the differences between daily life limitations and work limitations. This study aims at identifying the factors associated with limitations in personal and professional life in a population of working age suffering from shoulder pain.

**Methods:**

A sample of working age job seekers and workers with shoulder pain was drawn from the last general population cross-sectional French study on disability. Limitations were categorized depending on whether they related to daily life and/or work. The variables assessed were age, sex, state of health, activity restrictions, need for accommodation, and aggravating living conditions or aggravating working conditions. Separate Quasi-Poisson regressions were performed for each type of limitation.

**Results:**

The sample consisted of 795 individuals of which 33.7% had no limitation, 21.7% were limited in daily life, 6.0% at work, and 38.6% in both. Factors significantly associated with daily life limitations and work limitations and their computed Prevalence Ratios (PR) were the need for accommodation (PR = 2.16), activity restrictions (PR = 2.28), perceived poor health (PR = 2.42) and low income (PR = 1.64). Aggravating living conditions and aggravating working conditions were associated with daily life limitations (PR of 1.69 and 0.63 respectively).

**Conclusions:**

The present study identifies factors associated with disability in a population with shoulder pain. Further research should be carried out in order to study health-related periods of cessation of work.

## Background

The prevalence of shoulder pain ranges between 6.7 and 66.7% in the general population, depending on the age class, making it very common [1]. In the working population, 30% report daily shoulder pain during the previous year [2]. The pain can persist for months or even years in about a third of cases [3, 4]. Lasting aches, intense pain and severe functional limitations are noted as factors of poor prognosis regarding recovery and repercussion [5–7]. Lasting aches, intense pain, and an age over 55 are also associated with chronic shoulder pain [8]. In the workplace, pain intensity and middle age (ranging between 45 and 54 years for heavier work) are identified as having poor prognosis [6].

Shoulder pain can have deleterious effects on one’s professional career. In a French study, people with upper limb pain and musculoskeletal disorders were more likely to cease professional activity [9]. People with shoulder pain had lost between 1.8 and 8.1 years of work, over a 9-year follow-up period [10]. Moreover, when the work tasks put severe strain on the shoulder, disability can even persist after retirement, highlighting the need for studies concerning this issue [11].

Many factors are suspected of having a poor prognosis on the return to work or job retention, including activity limitations [12, 13]. Roe et al. highlighted the issues of common activity limitation at work, and of leisure and at home activities for people with shoulder pain [14]. Heavy work is associated with pain, functional limitations, and work limitations [6, 11], and even more for older workers [15]. However, it seems more difficult to characterize work disability situation for sedentary work or not heavy physical labor. Activity limitations can be overall or more specifically assessed [16]. To our knowledge, the consequences of shoulder pain on daily life and work activities have never been studied separately in the working population.

This work aims to assess the prevalence of daily life limitations and/or work limitations and their associated factors, among adults of working age suffering from shoulder discomfort (pain, stiffness, limitation of movements).

## Materials and methods

The Disability Health Survey was carried out between 2008 and 2009 by the National Institute for Statistics and Economic Studies (Institut national de la statistique et des études économiques, Insee) and the Department of Research, Studies, Evaluation and Statistics (Direction de la recherche, des études, de l’évaluation et des statistiques, DREES), and represents the last available cross-sectional survey on disability in the French general population. The objective of this survey was to study the frequency of disability and dependency according to the International Classification of Functioning, Disability and Health (ICF) [17], which provides a uniform and standardized language and a framework to describe health-related conditions. In the Disability Health Survey, disability was defined as the result of interactions of individual functional health status, and environmental factors. The survey included a “household” and “institutions” sections (according to the place of residence) with a specific questionnaire for each one.

The Household Health Disability Survey allowed to create a representative sample of the general population households, stratified according to a presumed level of disability and geographical areas. A preliminary questionnaire assessed the presumed level of disability. Stratification aimed at over-representing individuals with presumed severe level of disability. Trained investigators, with computer-assisted data collection, administered the household questionnaire between April and mid-July 2008. Collected variables included data on health, impairments, illnesses, functional limitations, activity restrictions (measured by several scales), education, employment, income, leisure, technical aids, human environment, accommodation, accessibility, and discrimination. These main topics were completed by 29,931 participants.

### Ethics

This study was planned as a research project. It was performed in collaboration with DREES. This study was declared of public interest by the Conseil National d’Information Statistique (CNIS) and was approved by appropriate ethics committee: the Commission Nationale de l’Informatique et des Libertés (CNIL, French law no. 78–17), then by State decision (arrêtés CE 2008–721 et 2009–1190). In 2007, according to the French law no. 78–17, written informed consent was not required for this type of study. The data used were taken from the National Disability-Heath survey, were anonymized prior to access and are available at: http://www.progedo-adisp.fr/enquetes/XML/lil.php?lil=lil-0459. All methods of the study were performed in accordance with the Helsinki Declaration.

### Studied sample

The household questionnaire included questions to identify severe joint problem (pain, stiffness, limitation of movement) which were used to filter participants with at least one impaired shoulder. Participants aged between 18 and 65, active workers or job seekers who had ever work, male and female, were included. Participants were excluded if there was a low quality of the responses to the questionnaire, if they had a history of working in an adapted environment, if they were not actively seeking a job or if they had missing data for the limitation variables.

### Studied variables

#### Dependent variable

The main outcome was a composite limitation criterion, assessed through two self-reported limitations that relate to the general state of health or disability, one concerning activities that most people are able to carry out (GALI - Global activity limitation indicator), and one related to work. The question: “For at least the past six months, to what extent have you been limited because of a health problem in activities people usually do?” from the Euro REVES module, provided the GALI indicator [18]. The question providing the work-related was formulated as follows: “Due to a disability or health problem, are you limited in the kind or amount of work you can do?” and was only asked to workers and active job seekers. In both cases, three choices were offered, depending on the presence and severity of the limitation (limited but not severely, severely limited, not limited at all). A composite limitation variable was created to fit the following categories, regardless of the severity: not limited, limitations in daily life activities only, limitations only at work, and finally, limitations in both.

#### Other variables

Socio-demographic variables such as age, sex and income per consumption unit were described using two classes each, respectively < 55 years vs. 55–65 years (corresponding to the fourth quartile and to the age close to retirement), male vs. female, and ≤ 1200 euros vs. > 1200 euros (corresponding to the fourth decile, close to the median income for a single-parent family in 2008, which was 1170 euros).

The state of health was described using bimodal parameters: perceived health status and activity restriction. Perceived health status was noted as good when the answer to the question “What is your general state of health?” was either “very good”, “good” and “average”, while “bad” and “very bad” were pooled and noted as bad. Activity was considered “restricted” upon the declaration of at least one restriction in ADL (Activities of Daily Living) or IADL (Instrumental Activities of Daily Living) [19]. ADL were assessed by several questions about degree of difficulties (some difficulty, great difficulty or cannot do it alone) for bathing, dressing, undressing, cutting food, pouring itself a drink, eating, drinking, using the bathroom, lying down in bed, getting out of bed, setting down from a chair alone, and getting up from a chair alone. A similar variable was constructed for IADL for the following situations: degree of difficulties for shopping, preparing meals, doing common household chores, doing occasional tasks and odd jobs, doing administrative processes, taking medication, moving around the different rooms, leaving their room or home, using a method of transportation, finding its way, and using a telephone or a computer alone. Other data collected included the feeling that living conditions or working conditions played a role or contributed to a worsening state of health, and the perceived need for an adaptation, or existence of working conditions or environment adaptation. The questions asked when a motor and mental impairment were declared were respectively: “Did your work conditions play a role in or contribute to worsening this motor (mental) problem?”, “Did your living conditions or another important event in your life play a role or contribute to worsening this motor (mental) problem?”. The question about the need for accommodation concerned people which didn’t receive disability work accommodation benefits: “Due to a disability or a health problem, was your work environment specially adapted, or in order to access a job, do you need special fittings, adapted work conditions or an adapted work environment?”. The motor or mental impairment were specifically asked as a consequence of living or working conditions, and the need for accommodation as a consequence of a health problem or disability.

### Analyzes

Qualitative variables were presented as raw frequency and weighted percentage in the descriptive analyzes. Missing data for non-dependent variables were due to the questionnaire structure which planned not to ask certain questions if: 1/ answers to a filter question was considered equivalent, 2/ the level of detail expected for each declared impairment was prioritized. These missing values represented less than 1–2% of case. They were considered as negative responses. When they really missing, the data were excluded from analyses.

Univariate Quasi-Poisson regression analyzes were performed between the dependent variables and the other variables to measure more precisely the strength of the associations due to the high prevalence of reported limitations in daily life and limitations at work (respectively 60.3 and 44.5%). The “no limitation” subgroup was considered as the reference. Sex, age, and income were studied through multivariate Quasi-Poisson regression analyzes. Stratified analyses were conducted for workers subgroup, looking for a job or not subgroups and low or high-income subgroups. Corrections and weighing were calculated in the Household Health Disability Survey to ensure that the collected data were representative of French households, notably regarding the probability of being interviewed and responding to the questionnaire, geographic sampling, and level of severity of disability. Sampling bias and non-response were corrected using these survey weights. Statistical analyses were performed using R software (Version 3.6.0, package “Survey”).

## Results

**Fig. 1:**
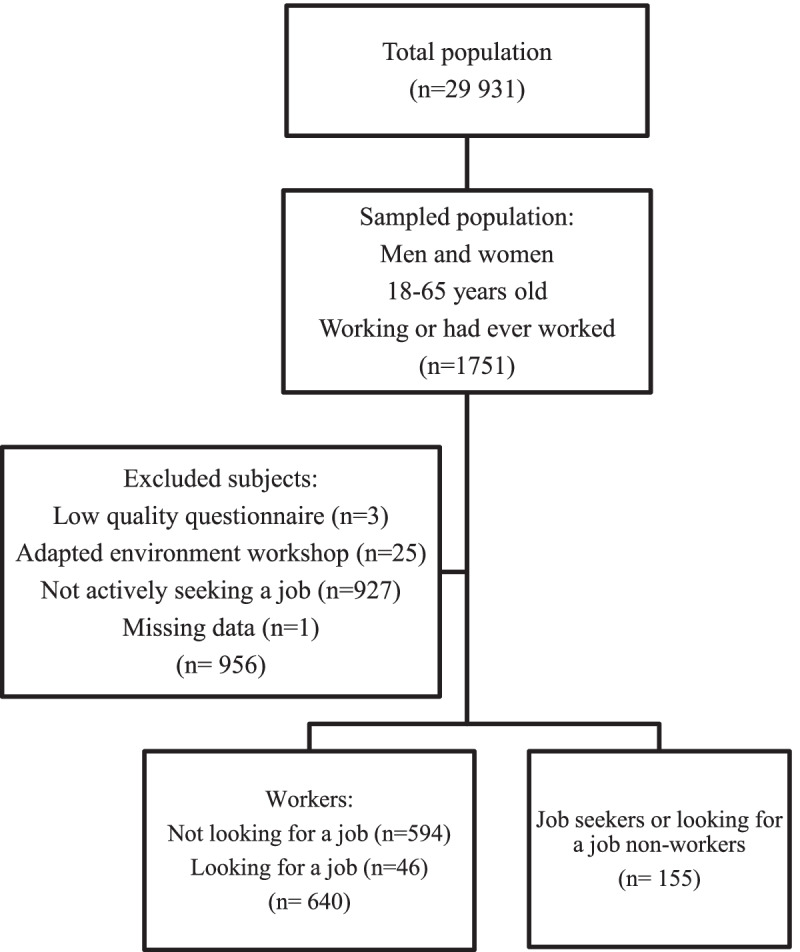
Flow chart

A flowchart in Fig. 1 shows participants’ inclusion in the study. Briefly, 1751 Household Health Disability Survey-responders corresponded to the working-age population with shoulder pain. The study sample was composed of 795 participants, including 640 workers (88.6%) and 155 job seekers (11.4%). A proportion of 201 participants declared to be looking for a job (17.5%), which involved all of the job seekers and 46 workers. Men and women accounted for 39.8 and 60.2% respectively. The average age was 47 years old years (SD: 9.4), varying between 18 and 65 years. The income per consumption unit was above 1200 euros in 61.5% of cases. The studied sample contained 33.7% of not limited people, 21.7% with daily life limitations, 6.0% with work limitations and 38.6% with both. A majority of person self-reported a good health status and 21.9% reported activity restriction. The participants reported, at the time of the survey, an average of 4.2 diseases (SD: 3.1) and 11.2 impairments (SD: 4.0), and 42.6% had at least one chronic disease. The cause of treated diseases was in particular joint and bone disease (49.1% of cases), heart disease (23.8%), neurologic disease (15.5%), psychiatric disease (9.7%), endocrine disease (8.7%) and cancer (1.5%). The need for accommodation concerned 16.3% of cases. Almost two thirds of people declared aggravating work conditions, and few declared aggravating living conditions.

Factors significantly associated with both limitations were the need for accommodation (PR = 2.16), activity restrictions (PR = 2.28), a poor health status (PR = 2.42), income lower than or equal to 1200 euros (PR = 1.64), and aggravating living conditions (PR = 1.60). Daily life limitations were associated with having a poor health status (PR = 2.25), activity restrictions (PR = 1.99), aggravating living conditions (PR = 1.69) and aggravating working conditions (PR = 0.63) (Table 1). Similar results were obtained within the workers subgroup, where aggravating working conditions becomes significantly associated with both limitations (PR = 1.56) (Table 2), and in the not looking for a job subgroup (PR = 1.72) (Table 3). In the looking for a job subgroup, only associations with health status, the need for accommodation and activity restrictions persist (Table 4).

**Table 1:** uni- and multivariate analyzes describing the factors associated to limitations (*n* = 795)

Variables		Total	No limitation	Daily life limitations	Prevalence ratio uni – 95% CI	Prevalence ratio multi – 95% CI	Work limitations	Prevalence ratio – 95% CI	Prevalence ratio – 95% CI	Both limitations	Prevalence ratio – 95% CI	Prevalence ratio – 95% CI
Age	18–54	583(74.9)	96 (78.6)	128 (74.3)	–	–	27 (70.0)	–	–	332 (72.7)	–	–
	55–65	212(25.1)	32 (21.4)	46 (25.7)	1.15 (0.74–1.80)	1.14 (0.73–1.80)	16 (30.0)	1.46 (0.61–3.48)	1.47 (0.63–3.42)	118 (27.3)	1.15 (0.87–1.53)	1.15 (0.89–1.49)
Sex	Male	295(39.8)	48 (44.7)	63 (43.9)	–	–	12 (28.5)	–	–	172 (35.0)	–	–
	Female	500(60.2)	80 (55.3)	111 (56.1)	1.02 (0.67–1.56)	1.01 (0.65–1.54)	31 (71.5)	1.83 (0.74–4.52)	1.64 (0.68–3.95)	278 (65.0)	1.21 (0.92–1.60)	1.20 (0.93–1.54)
Health status	Good	506(78.6)	122 (97.5)	129 (84.3)	–		40 (99.3)	–		215 (55.6)	–	
	Bad	289(21.4)	6 (2.5)	45 (15.7)	**2.25 (1.55–3.25)‡**		3 (0.7)	0.31 (0.04–2.10)		235 (44.4)	**2.42 (1.97–2.97)‡**	
Income	> 1200	420(61.5)	84 (73.4)	105 (68.3)	–	–	23 (61.2)	–	–	208 (47.2)	–	–
	<=1200	374(38.5)	44 (26.6)	68 (31.7)	1.16 (0.76–1.76)	1.17 (0.77–1.77)	20 (38.8)	1.59 (0.71–3.60)	1.53 (0.69–3.37)	242 (52.8)	**1.64 (1.27–2.11)‡**	1.63 (1.27–2.09)*
Accomodation	No	603(83.7)	125 (97.8)	157 (94.7)	–		36 (74.4)	–		285 (66.7)	–	
	Needed	192(16.3)	3 (2.2)	17 (5.3)	1.60 (0.88–2.90)		7 (25.6)	**5.73 (2.88–11.4)‡**		165 (33.3)	**2.16 (1.80–2.59)‡**	
Aggravating work conditions	No	259(35.9)	44 (38.3)	77 (57.3)	–		7 (16.3)	–		131 (25.0)	–	
	Yes	525(64.1)	82 (61.7)	91 (42.8)	0.63 (0.42–0.94)*		36 (83.7)	2.76 (0.84–9.07)		316 (75.0)	1.36 (0.99–1.88)	
Aggravating life conditions	No	665(87.9)	115 (94.6)	149 (86.1)	–		39 (97.1)	–		362 (81.7)	–	
	Yes	124(12.1)	12 (5.4)	24 (13.9)	1.69 (1.08–2.66)*		3 (2.9)	0.57 (0.10–3.27)		85 (18.3)	**1.60 (1.25–2.05)‡**	
Restriction	No	530(78.1)	120 (96.5)	137 (85.7)	–		33 (75.5)	–		240 (58.3)	–	
	Yes	265(21.9)	8 (3.5)	37 (14.3)	**1.99 (1.34–2.97)‡**		10 (24.6)	**4.56 (2.16–9.65)‡**		210 (41.7)	**2.28 (1.87–2.78)‡**	

‡*P* < 0.001; †*p* < 0.01; *p < 0.05

**Table 2:** uni- and multivariate analyzes describing the factors associated to limitations in the workers subgroup (*n* = 640)

Variables		Total	No limitation	Daily life limitations	Prevalence ratio uni – 95% CI	Prevalence ratio multi – 95% CI	Work limitations	Prevalence ratio uni – 95% CI	Prevalence ratio multi – 95% CI	Both limitations	Prevalence ratio uni – 95% CI	Prevalence ratio multi – 95% CI
Age	18–54	468 (74.8)	92 (79.7)	119 (72.1)	–	–	21 (69.6)	–	–	236 (72.1)	–	–
	55–65	172 (25.2)	31 (20.3)	41 (27.9)	1.28 (0.81–2.03)	1.28 (0.80–2.04)	14 (30.4)	1.57 (0.63–3.95)	1.58 (0.65–3.83)	86 (27.9)	1.23 (0.88–1.70)	1.22 (0.92–1.63)
Sex	Male	236 (40.2)	45 (44.9)	58 (43.3)	–	–	8 (25.8)	–	–	125 (35.9)	–	–
	Female	404 (59.8)	78 (55.1)	102 (56.7)	1.04 (0.67–1.63)	1.04 (0.66–1.64)	27 (74.2)	2.10 (0.75–5.84)	1.92 (0.72–5.13)	197 (64.1)	1.22 (0.88–1.68)	1.20 (0.89–1.61)
State of health	Good	438 (81.9)	117 (97.5)	119 (88.9)	–	–	33 (99.3)	–		169 (57.9)	–	
	Bad	202 (18.1)	6 (2.5)	41 (11.1)	**2.06 (1.30–3.27)†**		2 (0.7)	0.29 (0.04–2.25)		153 (42.1)	**2.62 (2.08–3.31)‡**	
Income	> 1200	372 (64.8)	83 (74.9)	100 (71.3)	–	–	22 (66.6)	–	–	167 (49.7)	–	–
	<=1200	267 (35.2)	40 (25.1)	59 (28.7)	1.12 (0.71–1.76)	1.14 (0.73–1.79)	13 (33.4)	1.41 (0.57–3.50)	1.36 (0.57–3.25)	155 (50.3)	**1.70 (1.27–2.27)‡**	1.70 (1.27–2.25)*
Accomodation	No	529 (86.3)	120 (97.8)	144 (94.9)	–	–	29 (73.7)	–		236 (71.0)	–	
	Needed	111 (13.7)	3 (2.2)	16 (5.1)	1.58 (0.82–3.05)		6 (26.3)	**5.97 (2.86–12.5)‡**		86 (29.0)	**2.28 (1.84–2.82)‡**	
Aggravating work conditions	No	198 (35.5)	41 (38.4)	71 (58.9)	–		3 (14.3)	–		83 (22.0)	–	
	Yes	432 (64.5)	80 (61.6)	83 (41.1)	0.59 (0.39–0.90)*		32 (85.7)	3.22 (0.78–13.3)		237 (78.0)	1.56 (1.06–2.27)*	
Aggravating life conditions	No	547 (90.6)	112 (96.4)	139 (89.0)	–		31 (96.8)	–		265 (84.4)	–	
	Yes	89 (9.4)	10 (3.6)	20 (11.0)	1.82 (1.13–2.93)*		3 (3.2)	0.90 (0.16–5.02)		56 (15.6)	**1.77 (1.35–2.33)‡**	
Restriction	No	445 (81.1)	116 (96.7)	127 (89.3)	–		26 (75.2)	–		176 (60.3)	–	
	Yes	195 (18.9)	7 (3.3)	33 (10.7)	**1.87 (1.18–2.96)†**		9 (24.8)	**4.87 (2.18–10.9)‡**		146 (39.7)	**2.49 (1.97–3.14)‡**	

‡*P* < 0.001; †*p* < 0.01; *p < 0.05

**Table 3:** uni- and multivariate analyzes describing the factors associated to limitations in the workers who are not looking for a job subgroup (*n* = 594)

Variables		Total	No limitation	Daily life limitations	Prevalence ratio uni – 95% CI	Prevalence ratio multi – 95% CI	Work limitations	Prevalence ratio uni – 95% CI	Prevalence ratio multi – 95% CI	Both limitations	Prevalence ratio uni – 95% CI	Prevalence ratio multi – 95% CI
Age	18–54	425 (73.5)	84 (77.8)	110 (71.2)	–	–	20 (69.4)	–	–	211 (71.2)	–	–
	55–65	169 (26.5)	30 (22.2)	40 (28.8)	1.23 (0.77–1.95)	1.23 (0.77–1.96)	14 (30.6)	1.43 (0.57–3.60)	1.48 (0.61–3.59)	85 (28.8)	1.19 (0.85–1.66)	1.19 (0.88–1.59)
Sex	Male	216 (38.9)	40 (42.4)	55 (43.0)	–	–	8 (25.9)	–	–	113 (35.2)	–	–
	Female	378 (61.1)	74 (57.6)	95 (57.0)	0.98 (0.63–1.55)	0.98 (0.62–1.54)	26 (74.1)	1.89 (0.68–5.25)	1.72 (0.64–4.62)	183 (64.8)	1.17 (0.84–1.63)	1.15 (0.85–1.56)
Health status	Good	403 (81.4)	108 (97.2)	111 (88.7)	–		32 (99.3)	–		152 (57.1)	–	
	Bad	191 (18.6)	6 (2.8)	39 (11.3)	**1.96 (1.23–3.12)†**		2 (0.7)	0.27 (0.03–2.06)		144 (42.9)	**2.58 (2.02–3.29)‡**	
Income	> 1200	352 (65.3)	78 (76.3)	96 (70.0)	–	–	21 (66.4)	–	–	157 (50.7)	–	–
	<=1200	241 (34.7)	36 (23.7)	53 (30.0)	1.21 (0.78–1.89)	1.24 (0.79–1.93)	13 (33.6)	1.50 (0.61–3.68)	1.45 (0.61–3.44)	139 (49.3)	**1.70 (1.27–2.29)‡**	1.70 (1.27–2.28)*
Accomodation	No	493 (86.6)	112 (98.9)	135 (94.8)	–		28 (73.6)	–		218 (71.1)	–	
	Needed	101 (13.4)	2 (1.1)	15 (5.2)	**1.97 (1.21–3.20)†**		6 (26.4)	**6.77 (3.68–12.4)‡**		78 (28.9)	**2.34 (1.91–2.87)‡**	
Aggravating work conditions	No	186 (36.4)	40 (40.7)	68 (60.6)	–		3 (14.4)	–		75 (20.7)	–	
	Yes	399 (63.6)	72 (59.3)	77 (39.4)	0.61 (0.39–0.93)*		31 (85.6)	3.44 (0.84–14.2)		219 (79.3)	**1.72 (1.15–2.57)†**	
Aggravating life conditions	No	514 (90.5)	104 (96.0)	131 (87.3)	–		30 (96.8)	–		249 (85.6)	–	
	Yes	77 (9.5)	9 (4.0)	19 (12.7)	**1.83 (1.16–2.87)†**		3 (3.2)	0.83 (0.15–4.65)		46 (14.4)	**1.67 (1.24–2.26)‡**	
Restriction	No	416 (80.7)	107 (96.3)	120 (89.3)	–		27 (76.4)	–		162 (59.9)	–	
	Yes	178 (19.3)	7 (3.7)	30 (10.7)	1.76 (1.09–2.82)*		7 (23.6)	**4.28 (1.88–9.75)‡**		134 (40.1)	**2.43 (1.91–3.10)‡**	

‡*P* < 0.001; †*p* < 0.01; *p < 0.05

**Table 4:** uni- and multivariate analyzes describing the factors associated to limitations in the looking for a job subgroup (*n* = 201)

Variables		Total	No limitation	Daily life limitations	Prevalence ratio uni – 95% CI	Prevalence ratio multi – 95% CI	Work limitations	Prevalence ratio uni – 95% CI	Both limitations	Prevalence ratio uni – 95% CI	Prevalence ratio multi – 95% CI
Age	18–54	148 (81.5)	12 (84.2)	18 (93.4)	–	–	7 (75.5)	/	121 (77.0)	–	–
	55–65	43 (18.5)	2 (15.8)	6 (6.6)	0.52 (0.10–2.75)	0.46 (0.09–2.39)	2 (24.5)	/	33 (23.0)	1.13 (0.71–1.81)	1.12 (0.67–1.88)
Sex	Male	79 (44.0)	8 (61.0)	8 (49.2)	–	–	4 (54.9)	/	59 (34.7)	–	–
	Female	122 (56.0)	6 (39.0)	16 (50.8)	1.32 (0.39–4.44)	1.68 (0.52–5.40)	5 (45.1)	/	95 (65.3)	1.39 (0.87–2.23)	1.38 (0.88–2.18)
Health status	Good	103 (65.1)	14 (100.0)	18 (56.9)	–		8 (99.2)	/	63 (51.1)	–	
	Bad	98 (34.9)	0 (0.0)	6 (43.1)	**3.43 (1.69–6.98)†**		1 (0.8)	/	91 (48.9)	**1.82 (1.25–2.65)†**	
Income	> 1200	68 (43.5)	6 (52.6)	9 (57.9)	–	–	2 (8.2)	/	51 (37.2)	–	–
	<=1200	133 (56.5)	8 (47.4)	15 (42.1)	0.88 (0.28–2.81)	0.76 (0.26–2.29)	7 (91.8)	/	103 (62.8)	1.21 (0.75–1.95)	1.19 (0.78–1.82)
Accomodation	No	110 (70.2)	13 (90.5)	22 (93.9)	–		8 (82.3)	/	67 (53.8)	–	
	Needed	91 (29.8)	1 (9.5)	2 (6.1)	0.74 (0.11–4.91)		1 (17.7)	/	87 (46.2)	1.57 (1.08–2.27)*	
Aggravating work conditions	No	73 (33.8)	4 (21.4)	9 (37.1)	–		4 (36.0)	/	56 (38.0)	–	
	Yes	126 (66.2)	10 (78.6)	14 (62.9)	0.66 (0.20–2.13)		5 (64.0)	/	97 (62.0)	0.80 (0.53–1.21)	
Aggravating life conditions	No	151 (76.0)	11 (84.8)	18 (78.2)	–		9 (100.0)	/	113 (70.3)	–	
	Yes	47 (24.0)	3 (15.2)	5 (21.8)	1.27 (0.35–4.67)		0 (0.0)	/	39 (29.7)	1.24 (0.81–1.90)	
Restriction	No	114 (66.0)	13 (97.6)	17 (63.1)	–		6 (66.2)	/	78 (53.7)	–	
	Yes	87 (34.0)	1 (2.4)	7 (36.9)	**2.87 (1.41–5.84)†**		3 (33.8)	/	76 (46.3)	**1.72 (1.23–2.42)†**	

‡*P* < 0.001; †*p* < 0.01; *p < 0.05

/: calculation not performed due to sample size

For people with income above 1200 euros, significant associations appeared for female sex and aggravating working conditions with both limitations. The rest of the associations were the same with higher PRs for the need for accommodation, poor health status, activity restrictions and aggravating living conditions. Only poor health status and activity restrictions remained associated with daily life limitation. The poor health status was also associated with work limitations (Table 5). As for the lower income subgroup, aggravating conditions were no longer associated with both limitations. Only activity restrictions were associated with daily limitations. Work limitations appeared associated with health status and aggravating living conditions (Table 6).

**Table 5:** uni- and multivariate analyzes describing the factors associated to limitations for the high-income subgroup (*n* = 420)

Variables		Total	No limitation	Daily life limitations	Prevalence ratio uni – 95% CI	Prevalence ratio multi – 95% CI	Work limitations	Prevalence ratio uni – 95% CI	Prevalence ratio multi – 95% CI	Both limitations	Prevalence ratio uni – 95% CI	Prevalence ratio multi – 95% CI
Age	18–54	292 (74.3)	61 (76.3)	76 (74.1)	–	–	12 (61.2)	–	–	143 (74.4)	–	–
	55–65	128 (25.7)	23 (23.7)	29 (25.9)	1.08 (0.60–1.91)	1.08 (0.60–1.91)	11 (38.8)	1.84 (0.61–5.52)	1.74 (0.61–4.95)	65 (25.6)	1.06 (0.67–1.67)	1.02 (0.66–1.58)
Sex	Male	160 (42.6)	35 (49.7)	37 (50.2)	–	–	8 (38.9)	–	–	80 (27.7)	–	–
	Female	260 (57.4)	49 (50.3)	68 (49.8)	0.99 (0.58–1.67)	0.99 (0.58–1.67)	15 (61.1)	1.47 (0.48–4.51)	1.34 (0.45–3.97)	128 (72.3)	**1.77 (1.16–2.70)†**	**1.77 (1.16–2.70)†**
Health status	Good	291 (84.4)	83 (100.0)	79 (84.0)	–		22 (99.2)	–		107 (60.6)	–	
	Bad	129 (15.6)	1 (0.0)	26 (16.0)	**2.99 (2.23–4.01)‡**		1 (0.8)	**6.08 (2.64–14.0)‡**		101 (39.4)	**3.24 (2.41–4.34)‡**	
Accomodation	No	334 (86.1)	82 (97.5)	92 (93.6)	–		18 (72.6)	–		142 (67.2)	–	
	Needed	86 (13.9)	2 (2.5)	13 (6.4)	1.65 (0.81–3.34)		5 (27.4)	**6.20 (2.41–16.0)‡**		66 (32.8)	**2.68 (2.00–3.60)‡**	
Aggravating work conditions	No	147 (38.6)	32 (42.5)	52 (60.5)	–		3 (11.1)	–		60 (22.2)	–	
	Yes	266 (61.4)	50 (57.5)	48 (39.5)	0.63 (0.37–1.06)		20 (88.9)	4.99 (0.75–33.1)		148 (77.8)	1.79 (1.06–3.04)*	
Aggravating life conditions	No	358 (88.8)	77 (96.5)	93 (90.1)	–		20 (95.5)	–		168 (76.1)	–	
	Yes	60 (11.2)	6 (3.5)	12 (9.9)	1.74 (0.94–3.22)		2 (4.5)	1.25 (0.19–8.19)		40 (23.9)	**2.25 (1.62–3.14)‡**	
Restriction	No	289 (81.2)	77 (95.6)	87 (86.6)	–		16 (72.5)	–		109 (59.1)	–	
	Yes	131 (18.8)	7 (4.4)	18 (13.4)	1.85 (1.02–3.33)*		7 (27.5)	**4.79 (1.74–13.2)†**		99 (40.9)	**2.79 (2.03–3.84)‡**	

‡*P* < 0.001; †*p* < 0.01; *p < 0.05

**Table 6:** uni- and multivariate analyzes describing the factors associated to limitations for low-income subgroup (*n* = 374)

Variables		Total	No limitation	Daily life limitations	Prevalence ratio uni – 95% CI	Prevalence ratio multi – 95% CI	Work limitations	Prevalence ratio uni – 95% CI	Prevalence ratio multi – 95% CI	Both limitations	Prevalence ratio uni – 95% CI	Prevalence ratio multi – 95% CI
Age	18–54	291 (76.1)	35 (85.0)	52 (76.6)	–	–	15 (83.9)	–	–	189 (71.2)	–	–
	55–65	83 (23.9)	9 (15.0)	16 (23.4)	1.33 (0.67–2.64)	1.33 (0.67–2.65)	5 (16.1)	1.07 (0.27–4.30)	1.09 (0.26–4.48)	53 (28.8)	1.24 (0.94–1.63)	1.24 (0.94–1.63)
Sex	Male	134 (35.1)	13 (30.9)	25 (28.8)	–	–	4 (12.3)	–	–	92 (41.6)	–	–
	Female	240 (64.9)	31 (69.1)	43 (71.2)	1.06 (0.51–2.18)	1.06 (0.51–2.189	16 (87.7)	2.66 (0.66–10.7)	2.66 (0.67–10.7)	150 (58.4)	0.87 (0.66–1.16)	0.88 (0.66–1.16)
Health status	Good	214 (69.2)	39 (90.8)	49 (84.6)	–		18 (99.5)	–		108 (51.1)	–	
	Bad	160 (30.8)	5 (9.2)	19 (15.4)	1.35 (0.63–2.89)		2 (0.5)	0.06 (0.01–0.49)*		134 (48.9)	**1.65 (1.27–2.13)‡**	
Accomodation	No	268 (80.0)	43 (98.9)	64 (96.9)	–		18 (77.1)	–		143 (66.2)	–	
	Needed	106 (20.0)	1 (1.1)	4 (3.1)	1.59 (0.67–3.76)		2 (22.9)	**5.05 (2.33–10.9)‡**		99 (33.8)	**1.64 (1.34–2.00)‡**	
Aggravating work conditions	No	112 (31.8)	12 (27.1)	25 (52.1)	–		4 (24.5)	–		71 (27.7)	–	
	Yes	258 (68.2)	32 (72.9)	42 (47.9)	0.56 (0.31–1.02)		16 (75.5)	1.11 (0.26–4.86)		168 (72.3)	0.99 (0.72–1.37)	
Aggravating life conditions	No	306 (86.5)	38 (89.7)	55 (76.9)	–		19 (99.6)	–		194 (86.8)	–	
	Yes	64 (13.5)	6 (10.3)	12 (23.1)	1.60 (0.82–3.12)		1 (0.4)	0.04 (0.00–0.48)*		45 (13.2)	1.08 (0.75–1.57)	
Restriction	No	241 (73.6)	43 (98.9)	50 (85.9)	–		17 (80.1)	–		131 (57.5)	–	
	Yes	133 (26.4)	1 (1.1)	18 (14.1)	**2.27 (1.50–3.44)‡**		3 (19.9)	**4.76 (2.11–10.7)‡**		111 (42.5)	**1.74 (1.39–2.17)‡**	

‡*P* < 0.001; †*p* < 0.01; *p < 0.05

## Discussion

This work highlights several factors associated with limitations in daily life and limitations at work in a sample of people suffering from shoulder pain: the need for accommodation, activity restrictions, perceived poor health, low income, aggravating working conditions and aggravating living conditions. The last two being associated with daily life limitations and working limitations, respectively. While activity restrictions were associated with daily life limitations and/or working limitations, health status was associated with daily life limitations and both limitations.

The results concerning the association with perceived health status [20, 21] and restrictions [12, 13, 22] are consistent with the literature studying disability using other indicators, such as return to employment, premature career exit, long-term absence, disability recognition, disability assessment questionnaire or work capacity. However, our results differ regarding other factors because associations will depend on disability situation. Concerning age and gender, across other studies, older age [13, 21–24] and female sex [13, 21, 23, 24] are identified as factors of poor prognosis for disability, although this may vary depending on the studied pathology [12]. These differences might be explained by the choice of the variable of interest in these studies, namely return to or prolonged absence from work, versus work or actively seeking a job in this study. The activity limitations in daily life studied in this does not appear to vary with age or gender [25]. Association between low social status and disability differs across the literature. Low socio-economic and educational level is sometimes associated with it [13, 24] and seems to depend on the disease [12]. We focused on money income per unit of consumption because it takes into account both the living standard and the household composition. In our results, lower incomes were associated with both limitations, except in the looking for a job subgroup. Unemployment, regardless its cause, could concerns people with limitations and without them equally in this subgroup. In the “not looking for a job” subgroup, it may probably reflect less skilled jobs or forced part-time work for people with both limitations, who have to continue to work despite their limitations. The need for accommodation was not specifically assessed so far, but similar results are described in studies addressing close issues, such as effectiveness of a work arrangement [13, 26], assessment of perceived work inability, part-time or precarious work as factors of poor prognosis for disability [12, 16, 27, 28]. In our study, the need for accommodation was associated with daily life limitations, only for the “not looking for a job subgroup”. These people could perceive limitation at workplace but not in carrying out work task because of the distribution of working time, commuting time, workplace access. Conversely, people can consider seeking a more adapted job when work demands do not change in a company.

Aggravating conditions in either daily life or at work are composite criteria, assessed by an open question. Specifications were provided inside the Disability Health study, through additional questions regarding financial difficulties, family issues, job loss, long-term unemployment, or inactivity, living or working difficulties due to physical suffering, exposure to nuisance, stress, or harassment either at home or at work. Physical difficulty (89.0%) and family issues (38.7%) were the most frequently reported, together with the alternate mention “other” (respectively 6.0 and 42.4%). Personal factors are insufficiently studied [12, 13], in addition to the social factors mentioned above and the important role of family life [16, 21, 29]. Aggravating working conditions may encompass physical and psychosocial occupational factors, which combine with musculoskeletal disorders during the onset of occupational disability [10, 13, 15, 21, 23, 30]. The type of occupation is also pointed out [12] but could not be studied in the present work due to missing data. The socio-professional category could have been a surrogate marker, but would have brought the need for pooling, thus limiting the power of the analysis.

Differences were found between daily life limitations and work limitations. Work is often synonym to constraining activities and compulsory tasks which may have negative effects on pain [31]. These results may reflect how disability impacts the work-life balance. Firstly, limitations lead to decreased performance and attendance at work [28]. Second, modifications in the nature of the undertaken tasks help maintain participation at home or at work [16]. This balance also depends on several factors such as the presence of children, fatigue, workload, control over work tasks and work limitations [29].

Our work provides a contrasting view of limitations in a working population. Limitations should be considered when assessing shoulder pain in routine practice to refer patient to specialized care into the workplace if necessary. Dissociating the consequences of shoulder pain on the daily life and work activities, in future research would allow to support and target actions for workers and job seekers in the workplace.

The focus on shoulder pain constitutes the main limit of this work. In the Household Health Disability Survey, the pathology causing the disability is unknown as well as the etiology of shoulder pain. Extrapolation of these results outside of the population suffering from shoulder pain without additional information regarding comorbidities is thus difficult. The aggravating conditions considered only relate to motor and mental disability. Overall, there was little change in the results upon withdrawal of people suffering from mental pathologies, except for a loss of significance for the association between aggravating working conditions and daily life limitations in the study population, and between aggravating working conditions and both limitations in the high-income subgroup. Further work needs to be undertaken in unemployed population, study power being another limiting factor of this work. Finally, this analysis, based on the latest available data on disability nationwide, despite using concepts that are still up-to-date (except for the age of retirement) would benefit from being reproduced with actualized information. Cross-sectional data allows to study complex and fluid conditions such as handicap, in a frozen state. The cross-sectional study design could lead to temporal bias. However, the way we formulated the questions on the need for accommodation and aggravating conditions allowed minimizing this bias. Finally, the use of separate, non-parsimonious simple Quasi-Poisson models may have minimized the strength of the associations but was necessary to compare these matched profiles. Multinomial logistic models resulted in the same trends.

## Conclusions

To conclude, the need for accommodation, activity restrictions, perceived poor health, low income, aggravating working conditions and aggravating living conditions were associated with daily life limitations and/or working limitations, with differences according to the limitations categories. Further work should also clarify the profiles found in the unemployed population, and especially just before quitting.

## Data Availability

The dataset (Handicap-Santé, volet Ménages (HSM) - 2008, INSEE [producteur], ADISP-CMH [diffuseur]) supporting the conclusions of this article is available on request at available at: http://www.progedo-adisp.fr/enquetes/XML/lil.php?lil=lil-0459

## References

[1] Luime JJ, Koes BW, Hendriksen IJM, Burdorf A, Verhagen AP, Miedema HS (2004). Prevalence and incidence of shoulder pain in the general population; a systematic review. Scand J Rheumatol.

[2] Roquelaure Y, Ha C, Leclerc A, Touranchet A, Sauteron M, Melchior M (2006). Epidemiologic surveillance of upper-extremity musculoskeletal disorders in the working population. Arthritis Care Res.

[3] Bodin J, Ha C, Petit A, Descatha A, Thomas T, Goldberg M (2014). Natural course of rotator cuff syndrome in a French working population. Am J Ind Med.

[4] van der Windt DA, Koes BW, Boeke AJ, Devillé W, De Jong BA, Bouter LM (1996). Shoulder disorders in general practice: prognostic indicators of outcome. Br J Gen Pract J R Coll Gen Pract.

[5] Bruls VEJ, Bastiaenen CHG, de Bie RA (2015). Prognostic factors of complaints of arm, neck, and/or shoulder: a systematic review of prospective cohort studies. Pain..

[6] Kuijpers T, van der Windt DAWM, van der Heijden GJMG, Bouter LM (2004). Systematic review of prognostic cohort studies on shoulder disorders. Pain..

[7] Kooijman MK, Barten DJA, Swinkels ICS, Kuijpers T, de Bakker D, Koes BW (2015). Pain intensity, neck pain and longer duration of complaints predict poorer outcome in patients with shoulder pain-a systematic review. BMC Musculoskelet Disord.

[8] Struyf F, Geraets J, Noten S, Meeus M, Nijs J (2016). A multivariable prediction model for the chronification of non-traumatic shoulder pain: a systematic review. Pain Physician.

[9] Sérazin C, Ha C, Bodin J, Imbernon E, Roquelaure Y (2013). Employment and occupational outcomes of workers with musculoskeletal pain in a French region. Occup Environ Med.

[10] Sirén M, Viikari-Juntura E, Arokoski J, Solovieva S (2019). Work participation and working life expectancy after a disabling shoulder lesion. Occup Environ Med.

[11] Descatha A, Teysseyre D, Cyr D, Imbernon E, Chastang JF, Plenet A (2012). Long-term effects of biomechanical exposure on severe shoulder pain in the Gazel cohort. Scand J Work Environ Health.

[12] Detaille SI, Heerkens YF, Engels JA, van der Gulden JWJ, van Dijk FJH (2009). Common prognostic factors of work disability among employees with a chronic somatic disease: a systematic review of cohort studies. Scand J Work Environ Health.

[13] Cancelliere C, Donovan J, Stochkendahl MJ, Biscardi M, Ammendolia C, Myburgh C (2016). Factors affecting return to work after injury or illness: best evidence synthesis of systematic reviews. Chiropr Man Ther.

[14] Roe Y, Bautz-Holter E, Juel NG, Soberg HL (2013). Identification of relevant international classification of functioning, disability and health categories in patients with shoulder pain: a cross-sectional study. J Rehabil Med.

[15] Bayattork M, Skovlund S, Sundstrup E, Andersen L (2021). Work limitations due to neck-shoulder pain and physical work demands in older workers: cross-sectional study. Int Arch Occup Environ Health.

[16] Beaton D, Bombardier C, Escorpizo R, Zhang W, Lacaille D, Boonen A (2009). Measuring worker productivity: frameworks and measures. J Rheumatol.

[17] World Health Organization. International classification of functioning, disability and Health (ICF). 2001; Geneva, WHO. https://www.who.int/classifications/icf/en/.

[18] Robine JM, Jagger C, Euro-REVES Group (2003). Creating a coherent set of indicators to monitor health across Europe: the euro-REVES 2 project. Eur J Pub Health.

[19] Katz S (1983). Assessing self-maintenance: activities of daily living, mobility, and instrumental activities of daily living. J Am Geriatr Soc.

[20] Vuistiner P, Luthi F, Erhart P, Scholz SM, Dériaz O (2015). Subjective perceptions as prognostic factors of time to fitness for work during a 4-year period after inpatient rehabilitation for orthopaedic trauma. Swiss Med Wkly.

[21] Slebus FG, Sluiter JK, Kuijer PPFM, Willems JHHBM, Frings-Dresen MHW (2007). Work-ability evaluation: a piece of cake or a hard nut to crack?. Disabil Rehabil.

[22] Valentin GH, Pilegaard MS, Vaegter HB, Rosendal M, Ortenblad L, Vaeggemose U (2016). Prognostic factors for disability and sick leave in patients with subacute non-malignant pain: a systematic review of cohort studies. BMJ Open.

[23] Abasolo L, Lajas C, Leon L, Carmona L, Macarron P, Candelas G (2012). Prognostic factors for long-term work disability due to musculoskeletal disorders. Rheumatol Int.

[24] Artus M, Campbell P, Mallen CD, Dunn KM, van der Windt DAW (2017). Generic prognostic factors for musculoskeletal pain in primary care: a systematic review. BMJ Open.

[25] Van Oyen H, Bogaert P, Yokota RTC, Berger N (2018). Measuring disability: a systematic review of the validity and reliability of the global activity limitations Indicator (GALI). Arch Public Health Arch Belg Sante Publique.

[26] Holland P, Collins AM (2018). “Whenever I can I push myself to go to work”: a qualitative study of experiences of sickness presenteeism among workers with rheumatoid arthritis. Disabil Rehabil.

[27] Ojala S, Pyöriä P (2019). Precarious work and the risk of receiving a disability pension. Scand J Public Health.

[28] van Schaaijk A, Nieuwenhuijsen K, Frings-Dresen M (2020). Work ability and percentage of hours worked related to limitations in patients with upper extremity musculoskeletal disorders: a cross-sectional cohort study. BMC Musculoskelet Disord.

[29] Gignac MAM, Cao X, Tang K, Beaton DE (2011). Examination of arthritis-related work place activity limitations and intermittent disability over four-and-a-half years and its relationship to job modifications and outcomes. Arthritis Care Res.

[30] Andersen LL, Fallentin N, Thorsen SV, Holtermann A (2016). Physical workload and risk of long-term sickness absence in the general working population and among blue-collar workers: prospective cohort study with register follow-up. Occup Environ Med.

[31] Tribian A, Vinstrup J, Sundstrup E, Jay K, Bös K, Andersen LL (2018). Physical activity during work and leisure show contrasting associations with fear-avoidance beliefs: cross-sectional study among more than 10,000 wage earners of the general working population. Scand J Pain.

